# Research on the Mechanical Properties and Microstructure of Modified Silt Sediment Geopolymer Materials

**DOI:** 10.3390/gels8120792

**Published:** 2022-12-02

**Authors:** Changming Li, Xiaoxiong Chai, Hui Liu, Haifeng Cheng, Dongyang Jia, Longfei Di, Songlin Qin, Yongbao Jin

**Affiliations:** 1 School of Civil Engineering and Communication, North China University of Water Resources and Electric Power, Zhengzhou 450045, China; 2 International Joint Research Lab for Eco-Building Materials and Engineering of Henan, North China University of Water Resources and Electric Power, Zhengzhou 450046, China; 3 Engineering and Technical Research Center of Levee Safety and Disease Control, Yellow River Institute of Hydraulic Research, Zhengzhou 450003, China; 4 Power Construction Corporation of China, Henan Wanshan Green Building Materials Co., Ltd., Luoyang 471000, China

**Keywords:** silted sediment utilization, alkali activation, geopolymer materials, compressive strength, microstructure

## Abstract

The treatment of silted sediment in the river is a global problem. The accumulation of waste sediment will lead to an adverse impact on the environment. In this paper, the silted sediment was reused to produce geopolymer composite materials via alkali-activated gelling modification. The effects of the modifiers of sodium silicate solution, quicklime, and Na_2_SO_4_ admixture, and the dosage of the slag, fly ash, and silica fume admixture, and curing conditions and age, on the compressive strength and microstructure of the geopolymer-modified sediment materials were studied. The crystalline phase and hydration products of the modified sediment geopolymer composites were analyzed by X-ray diffraction (XRD) and scanning electron microscopy (SEM) with energy dispersive spectroscopy (EDS), respectively. A compressive strength test was conducted to evaluate the mechanical properties of the composites. The results showed that the type and dosage of modifier, amount of mineral admixture additive, cure conditions, and cure age had significant effects on the mechanical properties of the composites. The effect of the addition of mineral admixture on the compressive strength of the modified sediment specimens was more noticeable than that of the modifier. The compressive strength of the geopolymer-modified specimens was greatly increased by the addition of mineral dopants. When 10 wt.% silica fume is added, the compressive strength reaches a maximum value of 33.25 MPa at 60 days. The SEM-EDS results show that the C-S-H gels and C-A-S-H gels were the main hydration products. The results indicate that river siltation sediment is an excellent raw material for geopolymer-modified materials. It is feasible to produce reliable and sustainable hydraulic engineering materials by using river sediment geopolymer-modified materials.

## 1. Introduction

In recent years, with the development of the economy and society, environmental protection has become the main concept of development in most countries in the world. High-energy consumption and high-emission materials are gradually being replaced by new green materials, and the shortage of traditional building materials is becoming more and more serious. The production of conventional Portland cement-based building materials accounts for approximately 6% of total global CO_2_ emissions [1,2].

Geopolymers are considered a new green building material with different applications in various fields such as coatings and adhesives [3], fiber composite production [4], decorative stone products [5], thermal insulation [6], construction materials, low energy tiles [7], waste encapsulation [8], thermal shock refractory materials [5], biotechnology [9], etc. Geopolymers can replace silicate cement, emit less CO_2_ compared to conventional concrete [10,11], and can reuse by-products and industrial wastes, which in most cases are difficult to dispose of [12,13]. Geopolymers have a faster setting time, but the setting rate allows transportation to the construction site [14]. As a result, they have received close attention from the scientific and industrial communities. Geopolymer materials are generated from raw materials rich in reactive silica-alumina under alkali-activated conditions, such as mixing fly ash, metakaolin, and other by-product materials with alkali solutions. Their mechanical properties and durability have been reported by many researchers to be comparable to conventional concrete [15,16,17,18,19]. For example, the maximum compressive strength of 113.8 MPa for geopolymer samples produced from loess and fly ash is much higher than that obtained with ordinary silicate cement (OPC) [20]. High-performance silicate cement (HPC) was compared with geopolymer concrete in terms of mechanical and thermal behavior and microstructural properties [21]. Regarding the compressive strength, the geopolymer concrete showed a faster setting time, reaching 15.0 MPa after only two hours.

Sediment consists mainly of solid particles visible to the naked eye that are produced by movement in the water flow or deposited by wind and gravity [22]. Sedimentation in rivers and reservoirs is a worldwide problem, and the huge volume of sediment will harm the ecological environment and human society in the form of debris flow, flooding, and pollution, and will also cause reservoir siltation, which will affect the flood capacity of rivers, navigation efficiency, agricultural production, and water conservation projects [23]. At the same time, the large amount of sediment accumulated after dredging will not only cause a waste of land resources, but will also cause serious ecological safety problems. Sediment consists of a variety of elements, such as organic matter and aluminosilicate minerals, such as quartz, feldspar, and clay, which makes it a great potential for construction materials and environmental protection [24,25,26,27,28,29]. If the modified sediment can be directly applied to a local river flood control project, it can, on the one hand, solve problems such as river siltation and, on the other, greatly reduce project costs. Previous studies have mostly used alkaline analytically pure reagents as modifiers to modify the sediment materials with alkali-activation, thus producing modified sediment geomaterials to replace some of the cement in concrete production, and have achieved good results [30]. Li G. [31,32] prepared a new composite material using the alkali-activated gelling modification method using Yellow River sediment as a raw material. The results revealed that the compressive strength of the modified samples increased with Ca(OH)_2_ dosage and curing age. The increase in strength was due to the formation of calcium silicate hydrate (C-S-H) gel and calcium aluminate hydrate (C-A-S-H) during the gelation modification process. These products fill the gaps between the composites, making the samples more compact. Pisha sandstone is one of the main sources of sediment in the Yellow River and has properties similar to sediment; Li C. [33,34] prepared geopolymer composite materials with Pisha sandstone as a raw material. It was found that the curing time, alkali dosage, and admixture content had significant effects on the mechanical properties of the composites, whose compressive strength could reach 20.3 MPa, and the main product of the gelling modification was the calcium silicate hydrate gel. Wang B. [35] prepared composites using Yellow River sediment as a raw material, and sodium silicate solution (water glass) and sodium hydroxide as modifiers, by the molding method. The compressive strength of the gelling material modified with Yellow River sediment increased significantly when the water glass dosing was 12 wt.% (M_s_ = 1.8) and the slag dosing was 40%, and the maximum compressive strength at 90 d could reach 53 MPa. Zheng L. [36] used different modifiers to gel-modify Yellow River sediment. The compressive strength of the Yellow River sediment-modified composites increased as the dosage of Ca(OH)_2_ increased, and the addition of NaOH resulted in a rapid increase in early strength. However, too high a dosage can lead to deterioration in compressive strength.

The above-mentioned studies show that sediment materials can be modified into geopolymer materials by alkali-activation to replace some cement production. However, current geopolymer modification studies do not consider comprehensively enough impact conditions, and it is difficult to refer to them in subsequent studies. If we can consider the impact of modifier type and admixture, external admixture type and admixture and various other conditions comprehensively, we can bring into play the great potential of geopolymer-modified sediment materials.

The purpose of this paper was to investigate the variation in compressive strength of modified silt-sediment geopolymers with the type and dosage of modifier and admixture, and the effect of curing conditions and curing age on the compressive strength, microstructure, hydration processes, and types of products produced. This leads to a better explanation of the mechanism related to modifying silt-sediment geopolymer materials.

## 2. Results and Discussion

### 2.1. The Effect of the Mole Ratio of SiO_2_ to Na_2_O of the Sodium Silicate Solution on the Compressive Strength

**Figure 1:**
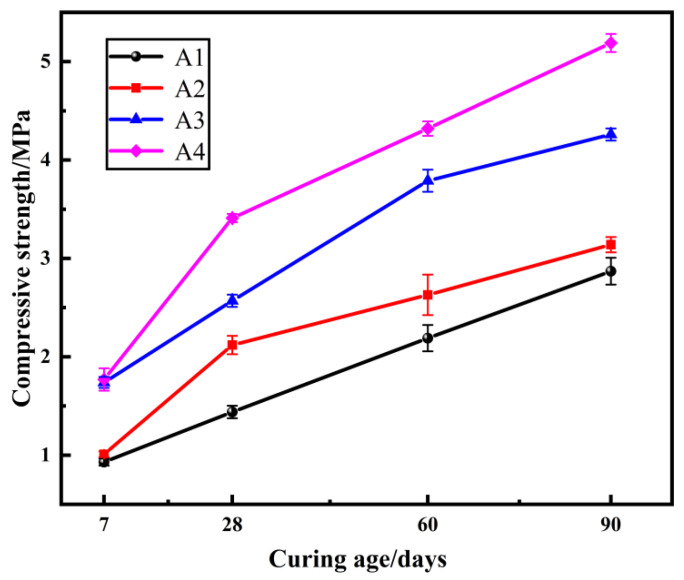
Strength of samples at different curing ages, varying the Na_2_O dosage.

The mole ratio of SiO_2_ to Na_2_O in the sodium silicate solution determines the alkalinity of the activator solution, which, in turn, affects the properties of the sample. Therefore, by choosing the mole ratios of SiO_2_ to Na_2_O in the sodium silicate solution as 3.0, 2.5, 2.0, and 1.5, their effects on the compressive strength of the samples were investigated. The compressive strength of the samples is shown in Figure 1.

From Figure 1 it can be seen that the compressive strength of the samples increases with the decrease in the mole ratio of SiO_2_ to Na_2_O in the sodium silicate solution, and the compressive strength of the samples increases with curing age. The Ca(OH)_2_ generated by the reaction of quicklime promotes the activation of the sediment and reacts with the reactive Si and Al components in the sediment to generate gelling substances. The hydration products C-S-H gel and C-A-S-H gel fill the gaps between the particles, making the sediment particles bond more tightly and improving the integrity of the samples; thus, enhancing the compressive strength of the samples. Samples of group A4 reached the highest strength of 5.19 MPa at the curing age of 90 days. However, this does not meet the strength requirements of flood control stone. If the strength of the samples were improved by only using NaOH to reduce the mole ratio of SiO_2_ to Na_2_O in the sodium silicate solution, the strength requirements may still not be achieved and it is not economical and satisfactory enough. Therefore, it is necessary to consider changing other conditions to improve the performance of the samples.

### 2.2. The Effect of Quicklime Dosage on Compressive Strength

**Figure 2:**
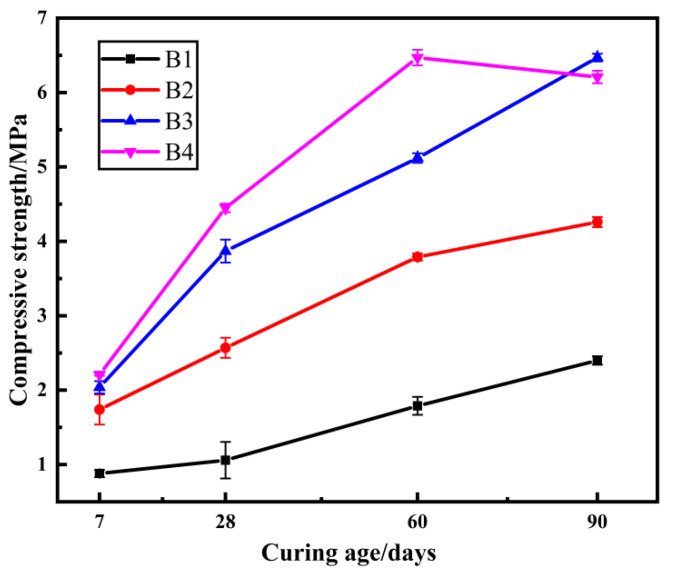
Strength of samples at different curing ages, varying the quicklime dosage.

The above tests found that it was not satisfactory to improve the compressive strength of the samples only by changing the mole ratio of SiO_2_ to Na_2_O in the sodium silicate solution. In addition, during the curing of the samples, it was found that the samples showed different degrees of surface alkali precipitation when the mole ratio of SiO_2_ to Na_2_O in the sodium silicate solution was 1.5. Therefore, subsequent tests were carried out with sodium silicate solution with a mole ratio of SiO_2_ to Na_2_O of 2.0, and the influence on the performance of the samples was investigated by changing the dosage of quicklime. The compressive strength of the samples is shown in Figure 2.

From Figure 2 it can be seen that the compressive strength of the samples increases with the increase in the dosage of quicklime. The compressive strength of samples in groups B1, B2, and B3 increased with increasing curing age, while the compressive strength of samples in group B4 with 10% quicklime admixture increased sequentially at curing ages of 7 days, 28 days, and 60 days, but decreased once the curing age reached 60 days. This is because the un-reacted Ca(OH)_2_ on the surface of the samples absorbed the CO_2_ in the air to generate CaCO_3_ due to the over-dosage of quicklime, which caused the volume of the samples to expand and small cracks to appear. Therefore, the dosage of admixture in the sediment geopolymer composites should not be too much, and excessive free OH^−^ residue in the samples will weaken the gel structure and damage the integrity of the material [37,38,39,40]. The compressive strength of the modified specimens reached a maximum of 6.48 MPa at the curing age of 60 d when the lime dosing was 10%, which was 24.86% higher than the maximum strength of 5.19 MPa in the above test of the effect of sodium silicate solution on the compressive strength of the modified specimens. Through the test of the effect of quicklime dosage, it was found that the addition of quicklime could effectively improve the early mechanical properties of the samples, and the optimal dosage was 5–9%, but the compressive strength at 90 days of curing age was less than 10 MPa, and it was necessary to consider changing other conditions to further improve the properties of the samples.

### 2.3. The Effect of Na_2_SO_4_ Dosage on Compressive Strength

**Figure 3:**
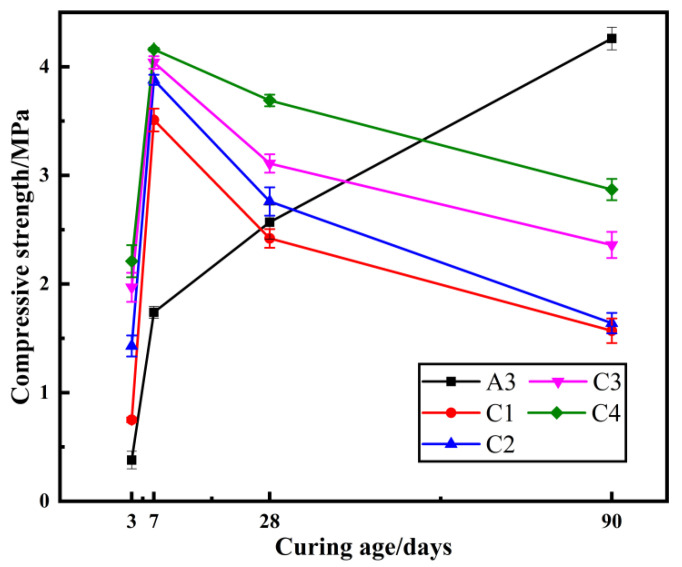
Strength of samples at different curing ages, varying the Na_2_SO_4_ dosage.

Based on the above test, Na_2_SO_4_ was added to investigate the influence of its content on the compressive strength law of the test samples. The compressive strength of the samples is shown in Figure 3.

From Figure 3 it can be seen that the compressive strength of the samples of group A3 without the admixture of Na_2_SO_4_ increases with increasing curing age. When Na_2_SO_4_ was added, initially, the compressive strength of the samples increased, and the compressive strength increased as the Na_2_SO_4_ dose increased. However, with the increase in the curing age, the strength decreased substantially, and some of the samples even appeared to disintegrate as the curing time was extended. The addition of SO_4_^2−^ caused this phenomenon by reacting with the Ca(OH)_2_ in the samples to generate CaSO_4_, which then reacted with hydrated calcium aluminate to produce calcium alumina (tri-sulfur-type hydrated calcium sulfur aluminate). The samples expanded in volume, causing them to crack at a later stage, and the strength first to increase and then decrease. Therefore, sulfate-based reagents are not suitable for use as gel-modifiers for sediment, and it was necessary to consider changing other conditions to further improve the properties of the samples. Mineral admixture is widely used in cement-based materials as an easily produced admixture. Therefore, mineral admixture was added in subsequent tests to effectively improve the compressive strength of the samples.

### 2.4. The Effect of Mineral Admixtures on Compressive Strength

**Figure 4:**
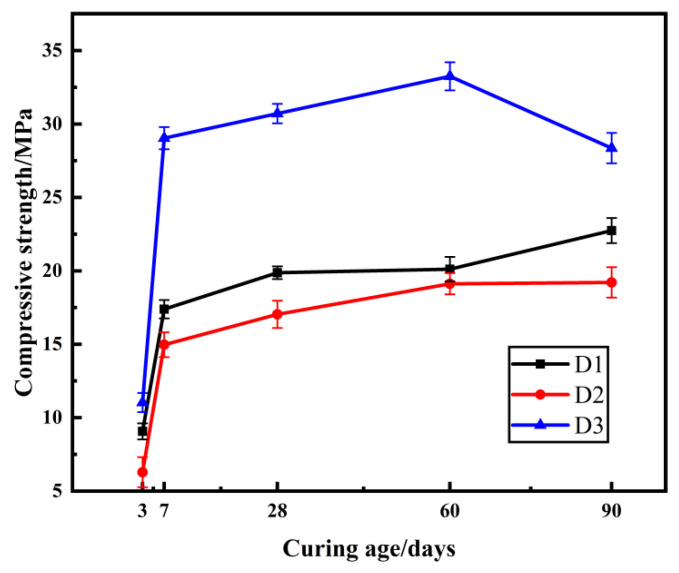
Strength of samples at different curing ages, with different mineral admixtures.

Additions of 10% slag, fly ash, and silica fume, respectively, were made to improve the properties of the samples containing 7% sodium silicate solution and 6% quicklime and study the effect of mineral admixture type on compressive strength. The compressive strength of the samples is shown in Figure 4.

From Figure 4 it can be seen that the three mineral admixtures of slag, fly ash, and silica fume can effectively improve the strength of the composite samples, and the modification effect of silica fume is better than that of slag and fly ash. The highest strength was achieved for the silica fume-modified samples of group D3, which reached 33.25 MPa at the age of 60 days after curing. Compared with the highest compressive strength of 6.48 MPa of the specimens in the earlier test without admixture modification, the compressive strength improvement effect increased significantly by 413.12%.The compressive strength of D1 and D2 group samples increased with the increase in curing age, and the increase was faster in the early stage and slower in the later stage, while the compressive strength of D3 group samples mixed with silica fume increased with the increase in curing age before 60 days and slightly decreased after 60 days. The compressive strength of silica fume-modified samples increased and then decreased with the increase in curing age because of the high activity of silica fume and its small particle size, the fast reaction of gel-modification, the rapid consumption of water in the samples, and the dry shrinkage in the dry air at the later stage of curing, which caused cracks in the samples and led to the decrease in compressive strength. Significantly, the 90-day curing age strength of the materials after the addition of mineral admixture is greater than 10 MPa, which meets the requirements of engineering applications.

### 2.5. The Effect of Curing Conditions on Compressive Strength

**Figure 5:**
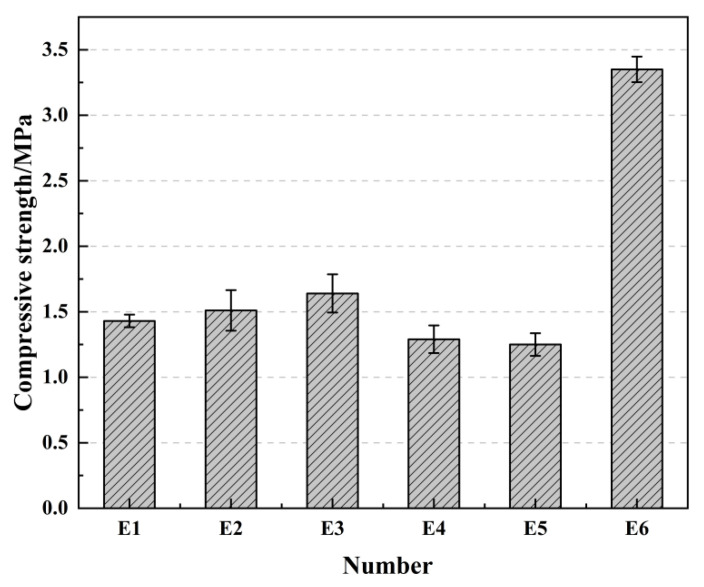
Compressive strength under different curing conditions.

From the above tests, it can be seen that the compressive strength of the mineral admixture-modified samples is better than that of the samples without admixture. For gelling materials, curing conditions also play a crucial role in their performance [41]. After studying the effect of the modifier type, the B2 group of samples was selected to study the effect of the curing conditions on the compressive strength of the samples. The curing conditions are shown in Table 1, and the compressive strength of the samples is shown in Figure 5. Natural curing is curing at room temperature, while standard curing for reference concrete is maintaining 20 ± 2 °C and humidity not less than 95% in a standard curing room.

From Figure 5 it can be seen that the curing conditions have an effect on the compressive strength of the samples. Compared to the compressive strength of the samples at the age of 28 days in natural curing, the compressive strength of the samples at the age of 28 days in standard curing and curing after immersion for 7 days increased, but the increase was smaller, while the compressive strength at the age of 28 days in immersion curing decreased. When comparing the compressive strength of E5 and E6, the compressive strength of natural curing for 28 days after high-temperature curing at 90 °C for 12 h is 3.35 MPa, which is 26.8% higher than the 1.25 MPa before natural curing, so the high-temperature drying curing method can significantly improve the compressive strength of the samples.

### 2.6. The Effect of Immersion Curing on Compressive Strength

**Figure 6:**
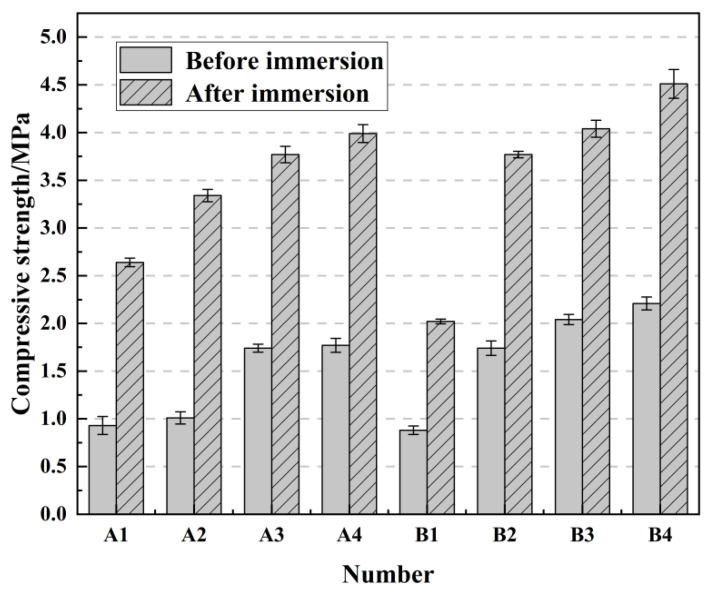
Change of compressive strength in 7 days curing age immersion curing.

**Figure 7:**
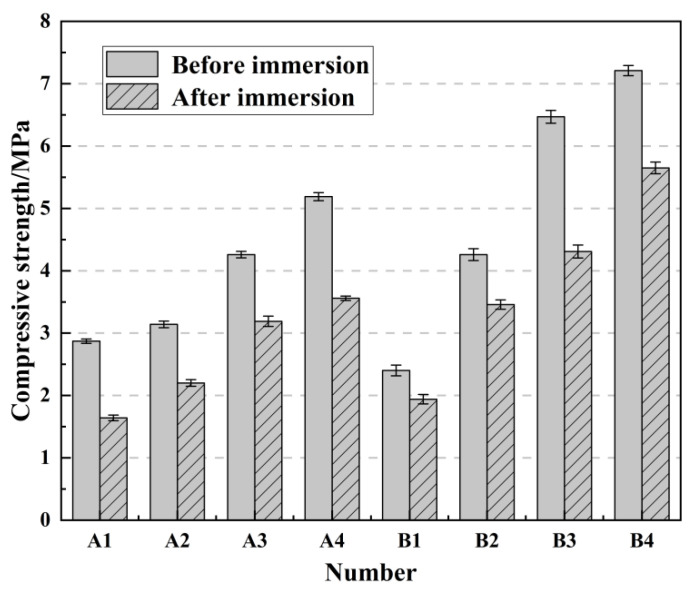
Change of compressive strength in 90 days curing age immersion curing.

The above test demonstrated that immersion curing has an effect on the compressive strength of the samples. In order to further investigate the effect of immersion curing on the compressive strength of long-age samples and short-age samples, the samples of groups A and B with 7 days and 90 days curing were taken as the objects of study, and each group of samples was immersed for 24 h and dried for 3 days, and the compressive strength was tested after the samples were dried and compared with the compressive strength before immersion to determine the relationship, and the strength changes are shown in Figure 6 and Figure 7.

The change in compressive strength of the samples in Figure 6 and Figure 7 shows that the strength of the samples in groups A and B at 7 days curing has increased through immersion. The largest increase in compressive strength of the samples was in the A2 group, from 1.01 MPa to 3.34 MPa, an increase of 203.69%. Conversely, the compressive strengths of the samples at 90 days curing were all reduced by immersion, and the largest reduction was from 2.87 MPa to 1.64 MPa in group A1, a reduction of 44.25%. It can be seen that early immersion can improve the compressive strength of the sample, bur prolonged immersion decreases the compressive strength. The reason for this phenomenon may be due to the fact that immersion in water in the early stage can accelerate the dissolution of active Si and Al components, accelerate their hydration reaction, and make the compressive strength of the sample increase rapidly; conversely, when the immersion is carried out in the later stage of curing, the hydration reaction is completed, and water will enter the microcracks inside the sample, which will then expand, weaken the bonding of the material inside the sample, and destroy its integrity, leading to the reduction in compressive strength. It is also possible that, when immersed in water for curing, there is too much water around the hydration products, which hinders their cohesion reaction and leads to a decrease in strength [42].

### 2.7. XRD Analysis

**Figure 8:**
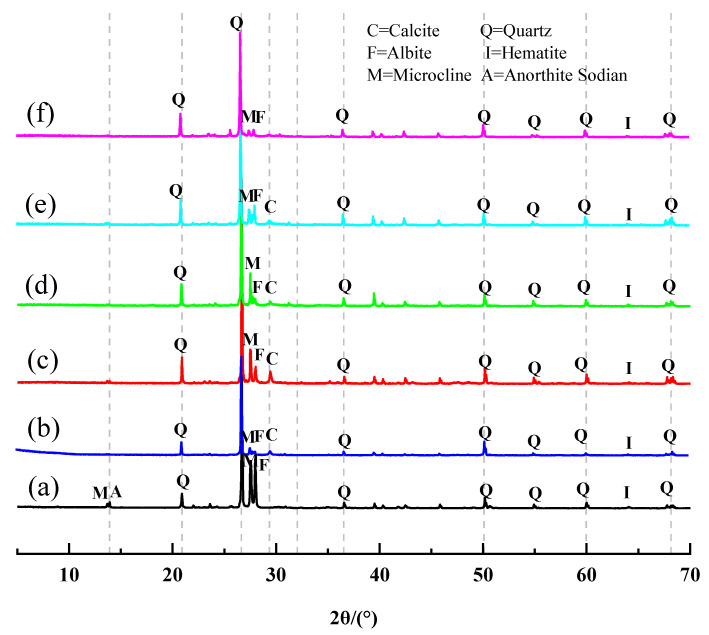
X-ray diffraction patterns of Xiaoluan River sediment and sediment geopolymer composites. (a) Xiaoluan River sediment, (b) A3: Ratio of SiO_2_ to Na_2_O in the sodium silicate solution is 2.0, (c) B3: Quicklime = 8 wt.%, (d) D1: Slag = 10 wt.%, (e) D2: Fly ash = 10 wt.%, (f) D3: Silica fume = 10 wt.%.

Figure 8 shows the XRD of the original and modified sediment samples. Some diffraction peaks have changed. Sharp peaks are observed at around 20.9° 2*θ* and 26.6° 2*θ* for the original sediment sample, indicating the presence of quartz (SiO_2_, PDF#46-1045), and albite (NaAlSi_3_O_8_, PDF#09-0466) appears at around 27.9° 2*θ*. The initial reaction of the modifier and mineral admixture-activated sediment seems to lead to the formation of C-S-H gels with carbonation in the C-S-H gels and the formation of CaCO_3_ at later maintenance ages [43]. From the analysis of the literature [44], the diffraction peaks at 20.9° 2*θ* and 50.1° 2*θ* are the characteristic diffraction peaks of the calcium silicate hydrate (Ca_1.5_Si_3.5_·nH_2_O). However, it was difficult to determine the diffraction characteristic peaks of C-S-H because they overlap with calcite peaks and quartz peaks. The presence of C-S-H gel needs to be further investigated using SEM-EDS. After geopolymer-modification, the microcline (KAlSi_3_O_8_, PDF#19-0932) peak at 27.5° 2*θ* and the albite (NaAlSi_3_O_8_, PDF#09-0466) peak at 27.9° 2*θ* became lower. The reduction of albite (NaAlSi_3_O_8_, PDF#09-0466) peak compared to microcline (KAlSi_3_O_8_, PDF#19-0932) peak in the quicklime- and slag-modified specimens is more obvious. Thus, it can be inferred that the sediments of the Xiaoluan River have potentially volcanic ash-active Si and Al.

### 2.8. Microstructural Analysis

**Figure 9:**
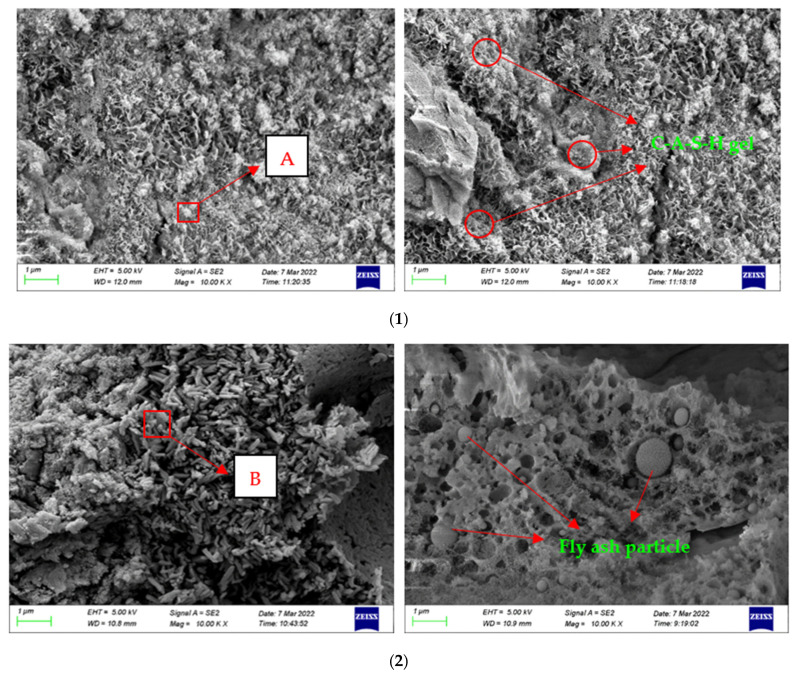

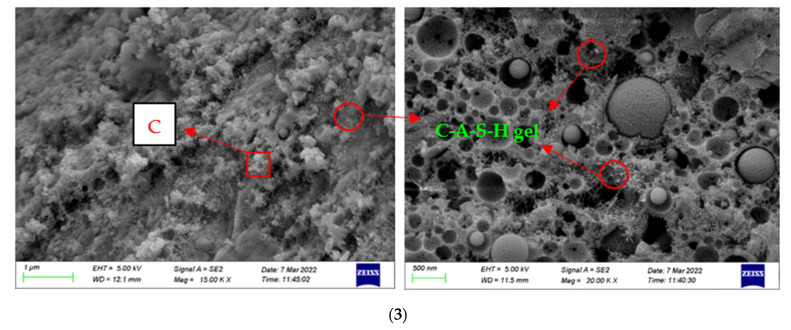
SEM image of modified composite gel material. (**1**) Slag-modified sample. (**2**) Fly-ash modified sample. (**3**) Silica fume-modified sample.

Figure 9 shows the scanning electron microscopy images of the composites after the modification of the mineral admixture. In order to determine the mineral composition of the new materials, we focused on the modified sediment particles observed in the SEM images of the materials, and acquired EDS energy spectrum to analyze the composition at the positions shown as “A,” “B,” and “C” in Figure 9. The results of the energy spectrum analysis are shown in Table 2. The scanned parts contain mainly carbon, oxygen, magnesium, aluminum, silicon, calcium, iron, etc. The weight percentages of Si and Ca elements confirm that C-S-H gel is one of the products in the composite. Inspection of the SEM images shows that, first, the gel-modified products are mainly glassy and amorphous particles, which contain a small number of impurities. Second, Figure 9(1) shows that the voids of the sediment particles are filled with some white material, which is clearly shown in location A. The white material fills the gaps between the particles and densely wraps the sediment particles. This gelatinous substance combines the stability of the sediment particles and the solid mass, helping to reduce the microcracks in the sample and thus improve its strength. Based on the above analysis, the white material may be the C-S-H gel, mainly produced by the mineral admixture under alkaline conditions. Although there were some cracks and holes in the internal structure, the samples were denser than before. The gel produced during alkali-activation plays an important role in filling the holes and improving the strength of the composite. The results show that the amount of C-S-H gel is small, which may be due to the low amount of mineral admixture and the fact that CO_2_ in the air can carbonize the C-S-H gel.

## 3. Conclusions

(1) River and lake sediment can be made into new geopolymer materials using the alkali-activated gel modification method. The sediment geopolymer materials produced have a maximum strength of 33.25 MPa, and the sediment geopolymer materials made according to this method have the potential to be used in water conservation projects such as rock preparation along rivers and lakes.
(2) Alkali dosage, mineral admixture type, and dosage, as well as curing condition and curing age, are significant factors affecting the mechanical strength of the composites. The strength of the samples will increase with increasing alkali dosage, but an excessive amount of alkali will have a negative impact on the compressive strength. Sulfate is not suitable as an alkali activator for geopolymer modification, and it will greatly reduce the durability of the modified material. Mineral dosage can significantly improve the early strength of the composites.
(3) High-temperature curing can accelerate the hydration reaction process and improve the reaction efficiency. Immersion curing can promote the growth of compressive strength of modified specimens in the early stage, while for long-age specimens immersion curing will weaken their compressive strength.
(4) The compressive strength of the samples was significantly enhanced after mixing with slag, fly ash, and silica fume. The XRD and SEM-EDS test results showed that the alkali-activated products were mainly flocculent or honeycomb geopolymer gels. The geopolymer gels were mainly amorphous hydrated calcium silicate (C-S-H) gels and hydrated calcium aluminate (C-A-S-H) gels, which then reacted with carbon dioxide to produce calcium carbonate, and the crystalline phase of the calcium carbonate in the composites was determined by XRD.

## 4. Materials and Methods

### 4.1. Raw Material

**Figure 10:**
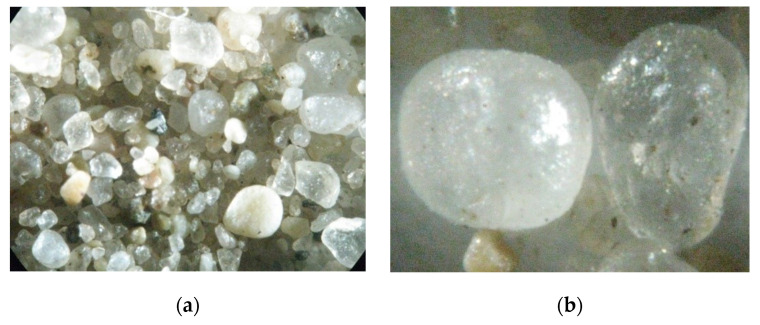
Particle morphology of the Xiaoluan River sediment. (**a**) Particle morphology at 300× magnification (**b**) Particle morphology at 1200× magnification.

The raw material for this experiment was taken from the Xiaoluan River in the western part of Weichang County, Hebei Province, China. Its particle morphology was observed using a SteRE0 Discovery V8 microscope from Zeiss, Germany, as shown in Figure 10. Referring to the subsequent XRD analysis, it can be seen that, at 300× magnification, the raw-material particles were mainly composed of crystalline particles with different grain sizes. At 1200× magnification, it was observed that the crystalline particles were irregular and translucent.

**Figure 11:**
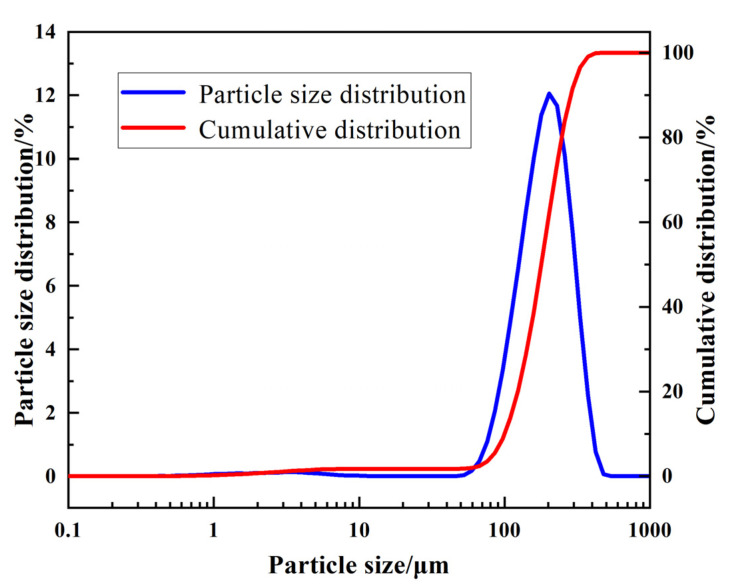
Particle size distribution curves of Xiaoluan River sediment.

The raw materials were mixed and then oven dried at 105 °C to a constant weight. After being cooled to room temperature, the particle size distribution (PSD) of the raw materials was measured with an NKL62000 type laser particle distribution meter from Jinan Nextech Analytical Instruments Co., Zhengzhou, China, and the fineness modulus of the Xiaoluan River sediment was also determined (Figure 11). As shown in Figure 11, it was found that the PDS of the raw materials was mainly between 150 and 500 μm, and was relatively concentrated and narrowly distributed. The chemical composition of the Xiaoluan River sediment was analyzed using an X-ray fluorescence spectrometer (XRF-1800) with Cu and Kα emission targets from Shimadzu Corporation, Japan (Table 3). From Table 3, it can be seen that the main components of the Xiaoluan River sediment are SiO_2_, Al_2_O_3_, CaO, Fe_2_O_3_, Na_2_O, and K_2_O. The content of these six oxides is approximately 95%.

**Figure 12:**
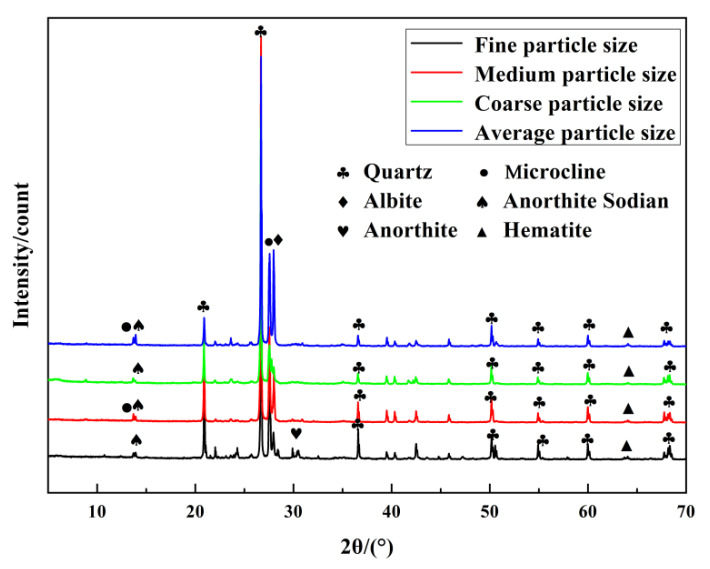
X-ray diffraction patterns of Xiaoluan river sediment.

Powdered samples of sediment were prepared using a mortar and pestle to ensure that the powdered samples were dry and could pass through a sieve of less than 20 μm. These samples were then used to fill a slide with a groove volume of 50 mm^3^. Then XRD data of the Xiaoluan River sediment with different grain sizes were recorded using a Bruker D8 Advance diffractometer (Cu target, k = 0.154 nm). The X-ray tube was operated at 40 kV and 40 mA. All scans were measured over an angular range of 5 to 70° 2θ at a rate of 2°/min and a step size of 0.02°. Figure 12 shows the XRD of the Xiaoluan River sediment. It can be seen that the main mineral components are quartz (SiO_2_, PDF#46-1045), anorthite (CaAl_2_Si_2_O_8_, PDF#41-1481), anorthite sodian ((Ca, Na)(Al, Si)_2_Si_2_O_8_), albite (NaAlSi_3_O_8_, PDF#09-0466), microcline (KAlSi_3_O_8_, PDF#19-0932), and hematite (Fe_2_O_3_, PDF#33-0664). The crystallinity of the quartz content is high, so its chemical composition is stable. The anorthite peaks are more significant in the fine-grained sediment, indicating that anorthite is mainly present in the fine-grained Xiaoluan River sediment.

### 4.2. Admixture

The modifiers used in the test were sodium silicate solution (the mole ratio of SiO_2_ to Na_2_O was 3), 99.9% pure analytical NaOH reagent, 99% pure analytical Na_2_SO_4_ reagent, and quicklime produced by Tianjin Comio Reagent Co., Tianjin, China. NaOH was used to adjust the mole ratio of SiO_2_ to Na_2_O of the sodium silicate solution. Since sodium silicate solution and quicklime were inexpensive and easily available materials and had relatively little environmental pollution, the modified new composite material could be applied to the manufacture of river basin flood-protection stones.

**Figure 13:**
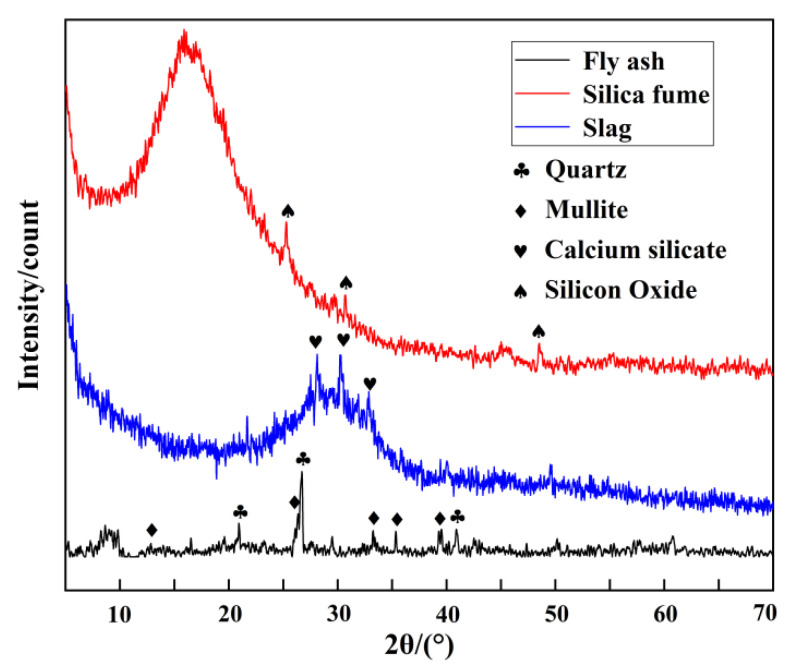
X-ray diffraction pattern of mineral admixture.

The S95 slag and silica fume used as mineral admixtures in this study were produced by Henan Borun Foundry Materials Co., Ltd., Zhengzhou, China, and fly ash was produced by Henan Gongyi Yuanheng Water Purification Materials Plant, Zhengzhou, China. If these industrial by-products, which are not easily disposed of, are used industrially for secondary use, they will not only bring economic and environmental benefits but will also solve the disposal problem of large amounts of waste [45,46]. Slag is made by reprocessing the floating molten slag on the surface of the iron from the ironmaking furnace [47]. Fly ash is a by-product of the combustion of pulverized coal in thermal power plants, and the growing industrialization in recent years had produced a large amount of fly ash [48]. Previous studies have shown that more than 750 million tons of fly ash are produced annually, but only about 17–20% was re-used [49]; silica fume belongs to the category of industrial waste utilization technologies, and results from the rapid oxidation and condensation precipitation of a large amount of SiO_2_ and silica fumes from mineral-heated electric furnaces during the smelting of ferrosilicon and industrial silicon. Silica fume is less dense and has to be processed using specialized encryption equipment [50,51]. The chemical composition of the mineral admixtures is shown in Table 4, and the XRD of the mineral admixture is shown in Figure 13. It can be seen that the main mineral component of fly ash is mullite (Al_6_Si_2_O_13_, PDF#15-0776) and quartz (SiO_2_, PDF#46-1045), the main mineral component of slag is calcium silicate (Ca_3_SiO_5_, PDF#16-0407), and the main mineral component of silica fume is silicon dioxide (SiO_2_, PDF#12-0708).

### 4.3. Casting, Curing and Testing

The sample was a cylindrical body D × H = 50 mm × 50 mm. The steel film pressure-forming method was used. The mold was a hollow cylindrical cylinder with an inner diameter of 50 mm, an outer diameter of 60 mm, and a height of 130 mm. The pressure bar was a solid cylinder with a diameter of 49.8 mm and height of 150 mm. The spacer was a solid cylinder with a diameter of 49.8 mm, height of 10 mm, and a cylinder with an outer diameter of 80 mm, an inner diameter of 55 mm, and height of 55 mm was used for sample demolding.

To account for the pressure formed by the samples, the density was set at 2100 kg/m^3^, and the mass of each raw material was calculated and weighed according to the material ratio. The volume of the sample was 98.125 cm^3^, so the mass of each sample was 206.06 g. After weighing and mixing, the material was mixed with a cement slurry mixer and then filled into the mold by drawing a horizontal line on the pressure bar 70 mm from the bottom, thus maintaining the height of the sample at 50 mm. After 5 min, the sample was demolded. After demolding, the samples were numbered and kept under the same conditions, sealed in plastic sealing bags, and placed in an oven at 90 °C for 12 h before curing, so that the compressive strength of the samples developed rapidly and reached a certain degree of compressive strength in a short period of time. The samples were kept in air for 3 days, 7 days, 28 days, and 90 days, respectively. The compressive strength of the samples was tested using an electronic universal testing machine with a range of 100 kN, and the displacement rate was 0.05 mm/min. The average value of three samples was taken as the compressive strength. The mineral content of the modified samples was determined using an X-ray diffractometer, and the microstructure of the modified samples was observed by scanning electron microscopy (SEM).

### 4.4. Mix Proportion Design

The mixed proportion of the samples is shown in the Table 5 The effect of various factors on the compressive strength of the samples was investigated by varying the composition of the mix and the admixture. Based on a preliminary test, the best solid-liquid ratio of 88:12 was selected considering the formation of the samples and preventing the mixed water from being squeezed.

## Figures and Tables

**Table 1:** Curing conditions.

Number	E1	E2	E3	E4	E5	E6
Curing conditions	Natural curing 28 days	Standard curing 28 days	Immersion 7 days after natural maintenance 28 days	Immersion curing 28 days	90 °C high temperature curing 12 h	Natural curing for 28 days after 90 °C high temperature curing

**Table 2:** Average molar ratio of basic elements at sample points A, B, and C in Figure 9.

Element	Sample A		Sample B		Sample C	
	Weight Percentage/wt.%	Atomic Percentage/at%	Weight Percentage/wt.%	Atomic Percentage/at%	Weight Percentage/wt.%	Atomic Percentage/at%
C	5.54	9.75	6.73	11.22	2.7	5.33
O	41.39	54.63	47.18	58.99	23.9	35.31
Na	2.1	1.93	5.54	4.82	2.26	2.32
Al	7.46	5.84	5.25	3.89	9.15	9.13
Si	20.96	15.81	15.02	10.73	47.23	40.13
Ca	19.27	10.17	17.75	8.88	11.9	7.03
Fe	2.03	0.77	1.39	0.52	1.32	0.6
Mg	1.25	1.1	1.14	0.95	1.54	0.15
Total	100		100		100	

**Table 3:** Chemical composition of the Xiaoluan River sediment (%).

Sample	Content of Each Component in the Experimental Material (%)									
	SiO_2_	Al_2_O_3_	K_2_O	Na_2_O	Fe_2_O_3_	CaO	MgO	TiO_2_	SO_3_	LOI
Xiaoluan River sediment	79.05	9.22	5.33	1.90	1.79	1.29	0.45	0.43	0.08	0.54

**Table 4:** Chemical composition of mineral admixtures.

Sample	Content of Each Component in the Experimental Material (%)									
	SiO_2_	Al_2_O_3_	K_2_O	Na_2_O	Fe_2_O_3_	CaO	MgO	TiO_2_	SO_3_	LOI
Slag	27.83	12.98	0.35	0.35	0.39	46.21	7.52	1.64	1.82	0.91
Fly ash	43.61	20.14	1.95	0.50	4.91	21.28	3.91	1.65	1.16	2.33
Silica fume	96.87	0.86	—	—	0.11	0.41	0.40	—	—	1.35

**Table 5:** Mixture proportion of samples.

Number	S/%	W/%	Sodium Silicate Solution/%	SiO_2_/Na_2_O	Q/%	Na_2_SO_4_/%	Mineral Admixture
A1	82	5	7	3.0	6	0	0
A2	82	5	7	2.5	6	0	0
A3	82	5	7	2.0	6	0	0
A4	82	5	7	1.5	6	0	0
B1	84	5	7	2.0	4	0	0
B2	82	5	7	2.0	6	0	0
B3	80	5	7	2.0	8	0	0
B4	78	5	7	2.0	10	0	0
C1	81	5	7	2.0	6	1	0
C2	79	5	7	2.0	6	3	0
C3	77	5	7	2.0	6	5	0
C4	75	5	7	2.0	6	7	0
D1	72	5	7	2.0	6	0	Slag 10%
D2	72	5	7	2.0	6	0	Fly ash10%
D3	72	5	7	2.0	6	0	Silica fume10%

S = Sediment, Q = Quicklime, W = Water, SiO_2_/Na_2_O was the mole ratio of the sodium silicate solution.

## Data Availability

Data obtained as described.
